# A job analysis of care helpers

**DOI:** 10.3352/jeehp.2012.9.2

**Published:** 2012-01-10

**Authors:** Su Jin Shin, Kyung-Sook Choi, Seungeun Jeong, Seulgee Kim, Hyeung-Keun Park, Jae Eun Seok

**Affiliations:** 1 Department of Nursing, Soonchunhyang University, Cheonan, Korea.; 2 Institute of Medical & Welfare Resources, Seoul, Korea.; 3 Department of Nursing, Andong Science College, Andong, Korea.; 4 Hamkkeguleum Health Cooperative, Seoul, Korea.; 5 Department of Health Policy and Management, Jeju National University School of Medicine, Jeju, Korea.; 6 Department of Social Welfare, Hallym University, Chuncheon, Korea.

**Keywords:** Long-term care, Job analysis, Care, Health personnel

## Abstract

The aim of this study was to examine the roles of care helpers through job analysis. To do this, this study used the Developing A Curriculum Method (DACUM) to classify job content and a multi-dimensional study design was applied to identify roles and create a job description by looking into the appropriateness, significance, frequency, and difficulty of job content as identified through workshops and cross-sectional surveys conducted for appropriateness verification. A total of 418 care helpers working in nursing facilities and community senior service facilities across the country were surveyed. The collected data were analyzed using PASW 18.0 software. Six duties and 18 tasks were identified based on the job model. Most tasks were found to be "important task", scoring 4.0 points or above. Physical care duties, elimination care, position changing and movement assistance, feeding assistance, and safety care were identified as high frequency tasks. The most difficult tasks were emergency prevention, early detection, and speedy reporting. A summary of the job of care helpers is providing physical, emotional, housekeeping, and daily activity assistance to elderly patients with problems in independently undertaking daily activities due to physical or mental causes in long-term care facilities or at the client's home. The results of this study suggest a task-focused examination, optimizing the content of the current standard teaching materials authorized by the Ministry of Health and Welfare while supplementing some content which was identified as task elements but not included in the current teaching materials and fully reflecting the actual frequency and difficulty of tasks.

## INTRODUCTION

The Long Term Care Insurance for the Elderly Act was enacted as a system where the nation and the society share the burden of supporting elderly people who are suffering from senile dementia or chronic degenerative diseases, and the policies for public support at the national level were implemented in July 2008. Since the Long Term Care Insurance for the Elderly Act was implemented, care helper system has been put into place and care helpers have provided the elderly who need long-term care with services such as physical assistance or housekeeping assistance.

Long-term care means providing health, social, and housing services to individuals whose functional capacities are chronically impaired [1]. Currently, care helpers are the principal official providers of long-term care services under the Long Term Care Insurance Act. In other words, care helpers are long-term care resources who provide physical and housekeeping assistance to elderly patients who have problems undertaking independent daily activities due to diseases that are common to the elderly population, such as dementia or strokes, in medical and long term care facilities for the elderly and community senior service facilities. Their principal aim is to improve the quality of life of the elderly through providing organized, professional services to long-term care recipients, including those aged 65 or above or those with geriatric diseases (stroke, dementia or Parkinson's disease) [2].

The Long Term Care Insurance Act for the Elderly defines care helpers as those official major resources providing long-term care services and long-term care resources who provide physical and housekeeping assistance to the elderly with problems in undertaking independent daily activities due to diseases that cause senility such as dementia or strokes in hospitals for the elderly, long term care facilities, and their home [3]. This study defines the job of care helpers as "the providers of physical and emotional support and housekeeping and daily activity assistance to the elderly with problems in undertaking independent daily activities due to physical or mental causes in long term care facilities and the client's home."

Stable operation of Long Term Care Insurance for the Elderly requires long-term care facilities satisfying the demand for long-term care and the establishment of a human resource infrastructure to secure quality services. To this end, the Korean government has newly introduced a national certified care helpers system to nurture long-term care professionals by strengthening the skills and knowledge of previous housekeepers and livelihood instructors [4].

A job analysis is a method and procedure for determining the duties, responsibilities, working conditions, job relations, and employee status of a position or among positions and offers an accurate picture of the job [5]. Therefore, to identify the practical characteristics of care helpers, the components of the job must be established, the qualifications required for successful performance specified, and the roles of care helpers defined through multidisciplinary cooperation among professionals related to senior care.

Care helpers, taking care of elderly care services and providing direct services at the center of home visit care services, need to empower the elderly to live healthy, stable lives with family members and relatives in a society by solving daily life problems and providing various necessary services while visiting elderly people's homes when they are alone and having the most frequent contact with them by their side [6]. In particular, the jobs of the newly introduced care helpers have become more diversified and comprehensive than those of the previous caregivers because the role of assistance with daily activities has been added to previous physical assistance [7].

However, many care recipients still recognize them as visiting housekeepers or housemaids [8], and care helpers are found to suffer the most severe stress when they are treated as housemaids while providing service. Therefore, the roles and responsibilities of care helpers must be clearly defined and realistic guidelines also need to be established accordingly. In addition, the number of long-term care facilities for the elderly and resources has been on the rise; therefore, to provide quality services, the most important thing is to improve the quality of care helpers, essential to the Long Term Care Insurance for the Elderly, and create the conditions in which this is possible.

Management of qualifications and education is important to nurture qualified human resources. In addition, the establishment of a legal framework, and the quality and capability of human resources, along with environmental support, play an important role in the actual success of the system. Particularly, though, it has been clearly established that nursing practices such as endotracheal suction, nasogastric feeding, and range of motion exercise, among care services for hospitalized patients, should not be performed by non-certified or unlicensed personnel. If they are undertaken by non-certified care helpers, it poses a risk of medical malpractice for problems such as foreign body aspiration, fracture, and infection [8,9]. Institutions for training care helpers have rapidly increased in number, and there have been some cases where people took advantage of the loophole in the system, granting a certification as long as education courses are completed without any testing or examination. To close the loophole, which can lead to the reckless granting of certification by recognizing qualifications based only on completion of an arbitrary training courses, without managing the content or quality of the training courses, efforts have been made for the nation to oversee a care helper certification.

The care helper certification exam was first administered in August 2010. However, to secure the validity of the exam, the concept, scope and requirements, and basis for the qualifying exam, need to be analyzed first; a job analysis is a prerequisite for securing the appropriateness of test items. Though several studies have been carried out on the job of care helpers, it is time to define and review the job though an analysis of the actual activities conducted in the field, as it has been three years since the system was implemented.

Therefore, the purpose of this study was to analyze the job of care helpers to determine its scope and, based on the result, to develop a formal job description and job requirements, ultimately providing grounds for a certification examination to establish a national certification system. The specific objectives were as follows:

1. To identify the duties, tasks, and task elements (concept and scope) of the care helper job
2. To verify the appropriateness and significance of tasks and task elements
3. To identify the frequency and difficulty of tasks and task elements

## MATERIALS AND METHODS

### Study design

This study aimed to examine the roles of care helpers through a job analysis. To do so, this study used the Developing A Curriculum Method (DACUM) to classify job content (duties and works) and a multi-dimensional study design to develop roles and a job description by investigating the appropriateness, significance, frequency, and difficulty of job content, as identified through workshops and cross-sectional surveys conducted for appropriateness verification.

### Study process

#### Identification of duties, tasks, and task elements

Preliminary items were identified through focus group interviews and the DACUM method was utilized as it is effective for identifying the objectives and content of education that lasts a relatively short period of time. A DACUM committee consisting of 8 to 12 panel members is usually used in DACUM method. This study selected eight DACUM committee members who agreed with the objective of this study and have experience either working as care helpers at the time of the study or educatingcare helpers. The ratio of care helpers to trainers was 5 to 3 in the first committee meetingand 2 to 5 in the second workshop. In the third round of the workshop, three of the care helpers reviewed the results of the second workshop, who were mostly the same people in each round.

#### Verification of the appropriateness and significance of the tasks and task elements

To verify the appropriateness and significance of job content identified through focus group interviews and DACUM workshops, an extended expert group, consisting of six scholars (2 from nursing science, 2 from social welfare, 1 from physical therapy and 1 from occupational therapy), was formed. The appropriateness and significance of the identified tasks and task elements were classified through a five-point scale and a job model was proposed.

#### Survey for job analysis

Surveys were carried out to verify the actual frequency and difficulty of the identified tasks and task elements in the field.

1) Sampling: Surveys were conducted with a total of 418 care helpers working in 48 nursing facilities and community senior service facilities across the country including the provinces of Seoul, Gyeongi, Gangwon, Chungchung, Gyeongsang, Jeolla, and Jeju. The number of samples was determined in proportion to the number of nursing facilities and community senior service facilities and the number of registered care helpers by region and a convenience sampling method was used to select facilities agreeing to participate in this study.

2) Data collection: Official letters were sent to the facilities to explain the purpose, intent, subject, and method of this study. Only among those facilities in which a facility representative understood the study purpose and agreed to participate, did each region's interviewers, who were educated about the guidelines and things to note, visit participants to fill out the questionnaire in person. The questionnaire consisted of tasks and task elements from the results of the DACUM workshop. A mail survey method was used for facilities which allowed the survey but were difficult to visit. The job components and the frequency of tasks were measured on a five-point scale (0, no; 1, 1 time a week or less; 2, 2 or 3 times a week; 3, once a day; 4, 2 to 3 times a day; 5, 4 times or above). To prevent the convergence toward the center that a five-point scale tends to produce, job components and the difficulty of tasks were measured by a four-point scale (1, very easy; 2, easy; 3, difficult; 4, very difficult). If the score was three or above, it was considered a difficult task.

3) Data analysis: The collected data were analyzed using PASW ver. 18.0 (SPSS Inc., Chicago, IL, USA). Descriptive statistics were utilized for determining frequencies, percentages, and means.

## RESULTS

### Job model

**Fig. 1:**
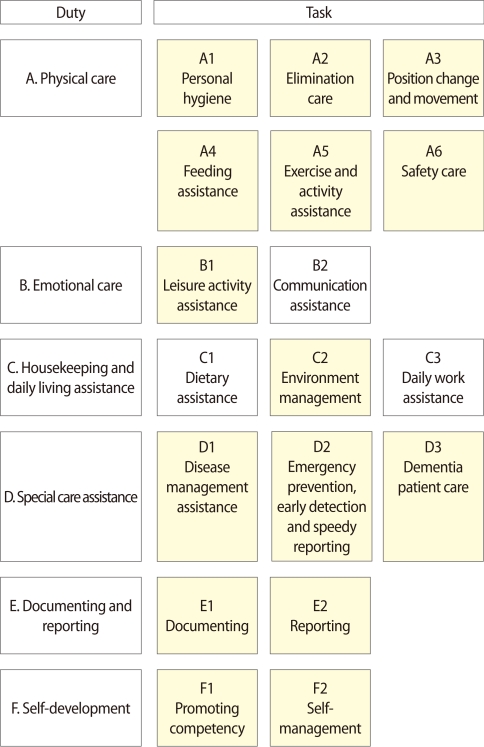
Job model. Tasks in boxes with yellow background are key tasks.

Six duties and 18 tasks were identified based on the job model deducted through the DACUM method. Figure 1 provides additional details. The key tasks were personal hygiene, elimination care, position change and movement, feeding assistance, exercise and activity assistance, safety care, leisure activity assistance, environment management, disease management assistance, emergency prevention, early detection and speedy reporting, dementia patient care, documenting, reporting, promoting competency, and self-management.

### Appropriateness and significance of tasks and task elements

Table 1 shows expert opinions on the appropriateness and significance of care helper's job tasks. On a five-point scale, most appropriateness items were surveyed as "appropriate" as they were 4.0 or above. However, there were opinions that personal counseling, grocery purchase assistance, and suction assistance, all of the three showing less than 3.5 points, were not appropriate. The significance of most tasks was found to be "significant" with points of 4.0 or above but dietary assistance (3.9) and daily work assistance (3.9) were relatively less significant.

### Frequency and difficulty of tasks and task elements

#### General characteristics of the surveyed participants

Table 2 shows the general characteristics of the surveyed participants. Among them, 67.9% of care helpers worked in nursing facilities while 32.1% worked in community long term care facilities. Male care helpers accounted for 6.1% while females were 93.9%. Most of the care helpers were in their 50s (41.8%), followed by those in their 40s (37.6%).

#### Frequency and difficulty of tasks and task elements

Tasks with three points or above are high-frequency repetitive activities which are done once a day or more (Table 3). By task, among physical care duties, elimination care, position change and movement assistance, feeding assistance, and safety care were identified as high frequency tasks. Leisure activity assistance was found to be a frequent activity among emotional care duties. Overall, position change and movement assistance (3.8), elimination care (3.5), leisure activity assistance (3.5), dementia patient care (3.4), safety care (3.3), and feeding assistance (3.2) were high-frequency repetitive tasks with frequency in that order. The documenting and reporting task and self-development task were scored as 1.3 to 2.4, which means they were carried out one to three times a week.

Task elements with high frequency were diaper change assistance (4.0), position change assistance (4.0), companion care (4.0), toilet care (3.9), bedsore prevention (3.8), fall prevention (3.8), elimination in the bed (3.7), observation (3.7), dental health care (3.6), wheelchair movement assistance (3.6), walking assistance (3.6), oral feeding assistance (3.6), bedding management (3.6), assistance with taking medicine (3.5), portable toilet assistance (3.4), observation of patients with dementia (3.4), handling of problematic behaviors of patients with dementia (3.4), assistance with cognitive activities for patients with dementia (3.4), perineal region cleaning assistance (3.3), face washing assistance (3.2), and cleaning (3.0).

The average difficulty of all tasks was 3.0, with distribution from 2.2 to 3.1. The most difficult tasks were emergency prevention, early detection, and speedy reporting. Other than these, safety management (2.9), dementia patient care (2.9), self-management (2.8), position change and movement assistance (2.7), elimination assistance (2.6), exercise and activity aid assistance (2.6), communication assistance (2.6), and daily work assistance (2.6) were found to be difficult.

Difficulty scores of task elements were distributed from 2.1 to 3.3. The most difficult task element was handling of death (3.3), followed by handling of suffocation (3.1), handling of convulsions (3.1), handling of falls and fractures (3.1), handling of other emergencies (3.1), handling of burns (3.0), handling of bleeding (3.0), handling of problematic behaviors of patients with dementia (3.0), assistance for cognitive activities of patients with dementia (3.0), bathing assistance (2.9), bedsore prevention (2.9), and handling of hypoglycemia (2.9).

Among physical care duties, elimination care, position change and movement assistance, exercise and activity assistance, and safety management were surveyed to be most difficult. Difficult task element were bathing assistance, perineal cleansing assistance, toilet assistance, elimination in the bed, portable toilet assistance, diaper change assistance, position change, wheelchair movement assistance, walking assistance, range of motion exercise assistance, occupational therapy assistance, physical therapy assistance, bedsore prevention, fall prevention, and restraint management. Communication assistance was found to be difficult among emotional care duties, and among job components, the most difficult ones were assistance for communication with outsiders and emotional support. Among housekeeping duties and daily living assistance with activities of daily living, daily work assistance was found to be most difficult.

The most difficult special care duties were emergency prevention, early detection, speedy reporting, and dementia patient care, and most job components related to those tasks were also found to be difficult. The most difficult task among recording and reporting duties and self-development duties was self-management. Among the task elements, stress management and health and safety management ware surveyed to be most difficult.

### Final job specification

The job summary of care helpers is "to provide physical, emotional, housekeeping, and daily activity assistance to the elderly with problems in undertaking independent daily activities due to physical or mental causes in long term care facilities and the client's home."

## DISCUSSION

This practical job analysis was a significant research project because it was a prerequisite to securing the validity of the care helper certification test.In the US and Japan, the job of care helpers is clearly distinguished from other medical occupations and they provide daily life aid services mainly under the supervision of nurses or social welfare workers. They are supposed to complete training courses in designated institutes [10,11]. In addition, they need to provide personal information, such as health records, identity verification, and a credit check, as prerequisites to working. On the other hand, there are no industry standards or job regulations for care services in Korea, and training is provided by private institutes. Therefore, it is necessary to take steps to improve the quality of education such as the development of continuing education programs.

Moreover, a state-run certification system was introduced in 2010, as there had been concerns over the decreasing quality of care helpers when certification was granted without control to anybody who completed a certain training courses regardless of educational background or age. However, supplementary measures are needed to improve the quality of caregivers and their services through training focusing on actual tasks and vocational ethics education in the future.

Based on the results of this study, two suggestions can be presented regarding the criteria for the care helper qualifying test put in place since August 2010. The first suggestion concerns standard teaching materials. Based on this job analysis and the standard teaching materials authorized by the Ministry of Health and Welfare, among basic care particulars, high-frequency tasks were position change and movement assistance, leisure activity assistance, elimination assistance and feeding assistance, and among special care particulars was dementia patient care. In addition, the most difficult tasks were emergency prevention, early detection, speedy reporting, safety management, dementia patient care, self-management, position change and movement assistance, elimination assistance, exercise and activity assistance, and communication assistance. Therefore, the proposed standard teaching materials suggest placing greater weight on basic care particulars and special care particulars that are needed to undertake tasks of high frequency or difficulty. The items on the certificate examination are categorized as introduction of care (17%), basic knowledge related to care (20%), basic care skills (46%), and special care particulars (17%).

The second suggestion is to establish a task-focused examination, making the best use of the content of the current standard teaching materials authorized by the Ministry of Health and Welfare while supplementing it with some additional content covering job components identified in the study but not included in the current teaching materials and fully reflecting the actual frequency and difficulty of tasks. To develop test items focusing on actual tasks, the second proposal suggests giving greater weight to high-frequency repetitive tasks (such as position change and movement assistance, personal hygiene assistance, elimination assistance, feeding assistance, leisure activity assistance, environment management, and dementia patient care) and difficult tasks with low frequency (such as emergency prevention, early detection, speedy reporting, exercise and rehabilitation assistance, communication assistance, competency improvement, and self-management). Therefore, the second proposal allocates 17% of test items for introduction of care, 13% for basic knowledge related to care, 52% for basic care skills, and 18% for special care particulars.

According to the results of this study, the existing standard teaching materials contain most of the job content identified through this job analysis but some additional tasks also need to be included. Those tasks include restraint management, play assistance, emotional support, occupational therapy assistance, physical therapy care, simple wound disinfection, expectoration assistance, and blood sugar level checking, which are not covered in the teaching materials but were identified as tasks in this study. This study also proposes that continuing education programs be developed for the existing care helpers who have already acquired certification.

## Figures and Tables

**Table 1:**
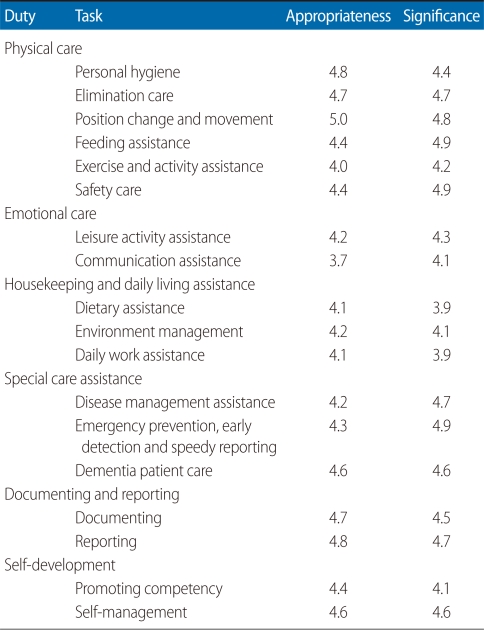
Appropriateness and significance of job content

**Table 2:**
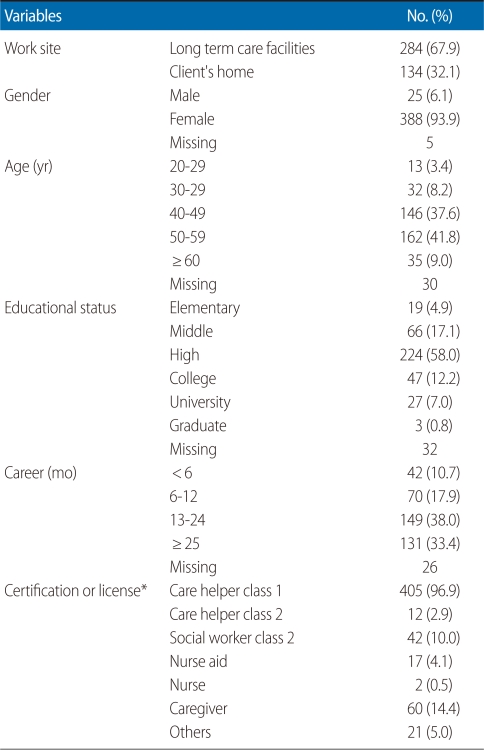
General characteristics of the participants (n=418)

^*^Multiple choice.

**Table 3:**
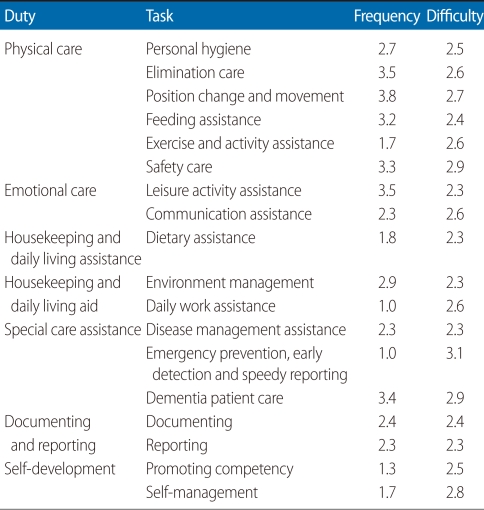
Frequency and difficulty of job content
